# 2.O. Workshop: Initial health assessments and catch-up vaccination for forcibly displaced migrants to Europe

**DOI:** 10.1093/eurpub/ckac129.113

**Published:** 2022-10-25

**Authors:** Organised by: St George's, Lancet Migration European Hub, ESGITM, Chair persons: Sally Hargreaves (UK), Ana Requena Mendez (Spain)

## Abstract

**Objectives:**

Recent crises in Ukraine, Afghanistan, Syria and other countries have, again, resulted in large populations of asylum seekers and other migrant groups arriving in Europe. However, European countries still grapple with questions about what level of health care should be offered to forcibly displaced people (emergency care, preventative care, multi-disease screening and catch-up vaccination?), and when and where in the migration trajectory provision should be made (should it be at borders, reception centres, once settled via the national health system, via specialist or routine services, or left to non-governmental organisations?), and what their subsequent level of right to access the mainstream health-care system should be. Countries do not have a uniform approach to the provision of health care for these populations, with some countries more inclusive than others, and wide discrepancies between evidence and implementation in policy and practice. In this workshop we will discuss current approaches, implementation, research and policy gaps, and models of good practice from the clinic to the community to ensure both the immediate and long-term health needs of these diverse mobile populations are met.

**Workshop plan:**

The workshop will start with 10-minute presentations by each of the 4 speakers (to include a short Q&A after each talk). This will then move into a 20-minute audience discussion centred seeking specific feedback on examples of successful interventions, good practice, and lessons learned across EU/EEA countries in delivering multi-disease and catch-up vaccination and holistic and inclusive healthcare approaches.

**Key messages:**

• Governments should develop clear short-term and long-term policy and evidence-led research strategies to ensure the equitable provision of health services.

• Strategies must include multi-disease and catch-up vaccination approaches, alongside meaningful access to mainstream health systems.

